# Emerging Roles of Myeloid-Derived Suppressor Cells in Diabetes

**DOI:** 10.3389/fphar.2021.798320

**Published:** 2021-12-16

**Authors:** Shiqi Wang, Qian Tan, Yayi Hou, Huan Dou

**Affiliations:** ^1^ The State Key Laboratory of Pharmaceutical Biotechnology, Division of Immunology, Medical School, Nanjing University, Nanjing, China; ^2^ Jiangsu Key Laboratory of Molecular Medicine, Nanjing University, Nanjing, China; ^3^ Department of Burns and Plastic Surgery, Nanjing Drum Tower Hospital, The Affiliated Hospital of Nanjing University Medical School, Nanjing, China

**Keywords:** diabetes, MDSC, recruitment, targeted therapy, diabetic complications, diabetic refractory wounds

## Abstract

Diabetes is a syndrome characterized by hyperglycemia with or without insulin resistance. Its etiology is attributed to the combined action of genes, environment and immune cells. Myeloid-derived suppressor cell (MDSC) is a heterogeneous population of immature cells with immunosuppressive ability. In recent years, different studies have debated the quantity, activity changes and roles of MDSC in the diabetic microenvironment. However, the emerging roles of MDSC have not been fully documented with regard to their interactions with diabetes. Here, the manifestations of MDSC and their subsets are reviewed with regard to the incidence of diabetes and diabetic complications. The possible drugs targeting MDSC are discussed with regard to their potential of treating diabetes. We believe that understanding MDSC will offer opportunities to explain pathological characteristics of different diabetes. MDSC also will be used for personalized immunotherapy of diabetes.

## Introduction

Diabetes has widespread incidence in almost every country and age group, which severely affects the worldwide economy and is regarded as an epidemic. According to the International Diabetes Federation, approximately 436 million patients worldwide had diabetes in 2019 and it was predicted that this number will exceed 700 million by 2045 (Saeedi et al., 2019). Diabetes is mainly divided into type 1 diabetes (T1D) and type 2 diabetes (T2D). T1D accounts for less than 10% of the total incidence of the disease, while T2D accounts for more than 90% of diabetic cases (Association, 2013). The immune system plays an essential role in the pathogenesis of diabetes: the insufficient central tolerance of thymus, too little variable number of tandem repeats (VNTR), quantitative and quality defects in Tregs lead to CD8^+^T cells attacking islet *β* cells, while inflammatory cytokines and recruitment of macrophages, B cells and CD4^+^T cells assist in attacking islet *β* cells, resulting in insufficient insulin secretion (Donath et al., 2019; Chen et al., 2021; Petrelli et al., 2021; Roep et al., 2021). At present, the drugs available on the market are mainly oral hypoglycemic drugs and insulin infusion, but there is no treatment for the basic pathological process that leads to *ß*-cell failure and destruction.

Although myeloid-derived suppressor cells (MDSC) have been reported to implicate in the incidence of diabetes, it has not been fully explored with regard to their interactions with diabetes (Whitfield-Larry et al., 2014; Wang T. et al., 2018; Hassan et al., 2018; Grohová et al., 2020). Herein, we reviewed the quantity, subtypes and activity of MDSC in the diabetic microenvironment. We also introduced the drugs targeting MDSC to delay the progression of this disease.

## Myeloid-Derived Suppressor Cells

MDSC have been defined for more than 30 years since the initial description of cancer patients (Buessow et al., 1984; Young et al., 1987; Seung and Schreiber, 1995). Immature bone marrow cells undergo activation, proliferation and differentiation into MDSC. MDSC are defined based on the CD11b^+^Gr-1^+^ phenotype in mice, whereas monocytic MDSC (M-MDSC) are defined as CD11b^+^Ly6C^hi^Ly6G^–^ cells with low side scatter. Polymorphonuclear MDSC (PMN-MDSC) that is granulocytic MDSC (G-MDSC), can be defined as CD11b^+^Ly6C^lo^Ly6G^+^ cells as determined by the high side scatter in the flow cytometry plot (Peranzoni et al., 2010; Damuzzo et al., 2015; Zhao et al., 2016). In view of the fact that the term PMN-MDSC is more capable of distinguishing from steady-state neutrophils (Bronte et al., 2016), we use the term PMN-MDSC in the following text. The markers on the surface of human MDSC are complicated. At present, human MDSC are defined by the expression of the common myeloid markers CD33 or CD11b and the lack of the marker of mature myeloid cells, such as HLA-DR, which is CD11b^+^/CD33^+^HLADR^−^. According to the molecular markers used, which are specific to the cluster, M-MDSC are defined as CD11b^+^/CD33^+^ HLA-DR^-^CD14^+^/CD15^−^/CD66b^−^ and PMN-MDSC as CD11b^+^/CD33^+^HLA-DR^-^CD14^−^/CD15^+^/CD66b^+^ (Gabrilovich and Srinivas, 2009; Zhao et al., 2016). In addition, early-stage MDSC (e-MDSC) lack molecular markers of any specific subtype, which are defined as CD11b^+^Gr-1^−^F4/80^−^MHCII^−^in mice (Zhang W. et al., 2018) and HLA-DR^–^CD33^+^Lin^–^(Lin: CD15, CD14, CD3, CD56, and CD19) in humans (Bronte et al., 2016). The latter is a newly defined type of MDSC, which is different from the other two subtypes.

The definition of MDSC depends on specific molecular markers and on their immunosuppressive ability. In particular, given that PMN-MDSC and neutrophils, M-MDSC and monocytes have the same source and differentiation pathway, they are almost indistinguishable in phenotype. In human peripheral blood, gradient centrifugation using 1.077 gl^−1^density can help isolate neutrophils and PMN-MDSC (Zhou et al., 2018). However, this method often leads to miscalculation due to the rise of activated neutrophils to low-density fraction and the preservation of specimens for too long and frozen damage. Specific surface markers of PMN-MDSC are still being studied. Mouse PMN-MDSC expressed higher levels of CD115 and CD244 than neutrophils (Youn et al., 2012), human PMN-MDSC expressed lectin type oxidized LDL receptor 1 (LOX-1) (Condamine et al., 2016). Although mouse M-MDSC also expressed F4/80, M-MDSC can still be isolated from macrophages and dendritic cells by detection of low levels of both MHC class II and the dendritic cell marker CD11c. Human M-MDSC expressed higher S100A8/A9 and lower HLA-DR (Bronte et al., 2016; Kwak et al., 2020).

The specific tests that can verify the inhibitory ability of MDSC on T cells have become the gold standard. In mouse tests, the immunosuppressive ability was determined by measuring T-cell proliferation or inhibition of interferon (IFN)-γ production following MDSC culture with antigen-specific and antigen-nonspecific T cells. In human tests, the validation was divided into three groups as follows: Following the addition of candidate MDSC population, the detection of T cell proliferation or IFN-γ production was performed; following removal of the MDSC population, the measurement of T cell proliferation was performed and following allotransplantation, the measurement of T cell proliferation or IFN-γ production was performed (Bronte et al., 2016). During the process of assessing the immune ability, M-MDSC exhibited the highest immunosuppressive function, while PMN-MDSC had the weakest (Zhao et al., 2016).

MDSC play different roles in various pathological processes by direct cell contact and secretion of immunosuppressive factors during various pathological processes, including tumor progression, infection and autoimmune diseases. MDSC are prominent in immunosuppression, inflammation, and angiogenesis, which are also the characteristic features of diabetes. However, their role in diabetes has not been fully explored.

## Number of MDSC in Diabetes

MDSC are immature bone marrow cells in healthy individuals that account for 0.5–1% of peripheral blood HLA-DR^-^ cells (Whitfield-Larry et al., 2014; Hassan et al., 2018). Recent studies have demonstrated that the total numbers of MDSC increased in T1D and T2D subjects (summarized in Table 1).

**TABLE 1:** Quantity changes of MDSC and subsets of MDSC in the environment of diabetes.

		MDSC	Subtypes	Reference
Type 1 diabetes	NOD mice	Expanded in peripheral blood and secondary lymphoid organ	M-MDSC increased significantly	Whitfield-Larry et al. (2014)
	NOD mice	Increased in bone marrow and peripheral blood	—	Yin et al. (2010)
	NOD mice	Decreased in islets	—	Fu et al. (2012)
	NOD mice	—	12-week-old showed a decrease in M-MDSC and an increase in PMN-MDSC compared to 4-week-old before the onset	Qi et al. (2020)
	STZ mice	Increased significantly on day three after STZ injection and then remained higher than in the control group until the last day (day 24) of observation and remained stable at about twice the percentage of the control group in peripheral blood	—	Venneri et al. (2015)
	STZ mice	Increased in peripheral blood (36.5 vs. 20.5%, *P* = 0.07)	—	Hsieh et al. (2018)
	STZ mice	Decrease at the fourth week in the bone marrow	—	Furman, (2015)
	STZ mice	The number of MDSC return to normal at the sixth week	6 weeks after STZ-induced diabetes in mice, PMN-MDSC accounted for a large proportion in bone marrow, but the G/M ratio decreased, although there was no statistical significance	Kim et al. (2018)
	STZ mice	Increased in the spleen, bone marrow, kidney, and PLN 3 weeks after STZ treatment	—	(Gao et al., 2013; Hsieh et al., 2018)
	STZ mice	—	The proportion of PMN-MDSC in bone marrow slightly decreased while the proportion of M-MDSC slightly increased, with statistical differences	Li et al. (2021)
	STZ mice	—	The ratio of M-MDSC decreased in PLNs 15 days after STZ treatment	Carlos et al. (2017)
	T1D patients (aged 11–46 years), *n* = 23 vs. healthy volunteers (aged 12–59 years), *n* = 21	Significantly increased in the peripheral blood of patients with T1D, and most of them were M-MDSC	M-MDSC significant increased in the spleen and peripheral blood but decreased in pancreatic lymph nodes (PLNS) 15 days after STZ treatment	Whitfield-Larry et al. (2014)
	T1D patients, *n* = 30 vs. non-diabetic patients, *n* = 30	MDSC in nucleated cells increased by 0.72 ± 0.24 (non-diabetic patients) vs. 4.4 ± 2.07 (diabetic patients)	A slight decrease in the M-MDSC of CD14^+^ cells (99.3 ± 0.3 vs. 96.5 ± 3.02) and a significant increase in the PMN-MDSC of CD14^−^ cells (0.62 ± 0.33 vs. 3.98 ± 3.0) in peripheral blood	Hassan et al. (2018)
	T1D patients, *n* = 65 vs. their high-risk relatives (diabetes-related antibody positive), *n* = 21 vs. healthy volunteers, *n* = 24	The number of MDSC, especially the subgroup M-MDSC, increased significantly. M-MDSC in the group with HbA1c >7.5% is significantly higher	—	Grohová et al. (2020)
Type 2 diabetes	db/db mice	MDSC increased in spleen cells (4.5 vs. 2.3%) and peripheral blood (23 vs. 11%) (compared with that in the healthy control group)	—	Wang et al. (2018a)
	ob/ob mice	MDSC in bone marrow did not change. The number of MDSC in the spleen, fat, and liver of peripheral organs increased significantly	—	Xia et al. (2011)
	ob/ob mice	Bone marrow-derived MDSC produced significantly less CFU G and significantly more CFU M	—	Mahdipour et al. (2011)
	T2D (30–55 years old), *n* = 24 vs. healthy volunteers, *n* = 22	Increase in the proportion of MDSC in peripheral blood	—	Wang et al. (2018a)
	T2D, *n* = 22 vs. no-diabetes patients, *n* = 21	The median frequency of MDSC in peripheral blood was 1.5 and 1%, respectively, and the difference was statistically significant	—	Fernández-Ruiz et al. (2019)
	T2D, *n* = 80 vs. healthy volunteers, *n* = 11	The proportion of MDSC in PBMC was higher in T2D patients than in healthy subjects (median, 6.7 vs. 2.5%); among this study, PMN-MDSC accounted for 96% of MDSC	—	Islam et al. (2020)
	T2D, *n* = 19 vs. obese normal glucose volunteers, *n* = 18	The number of M-MDSC increased in peripheral blood (*P* = 0.048)	The number of M-MDSC in the peripheral blood of obese T2D patients was higher than that of obese non-T2D patients	Friedrich et al. (2019)

NOD mice, (non-obese diabetic mice); STZ mice, (streptozotocin-induced diabetic mice); PMN-MDSC (polymorphonuclear myeloid-derived suppressor cells); M-MDSC, (monocytes myeloid-derived suppressor cells); T1D, (type 1 diabetes); T2D, (type 2 diabetes).

In T1D, the non-obese diabetic (NOD) mouse model and STZ mouse model are commonly used in basic experiments (Busineni et al., 2015; Chen et al., 2018). Compared with prediabetic (10–14 week old) NOD mice, newly diabetic NOD mice had an MDSC (mainly M-MDSC) expansion in bone marrow, peripheral blood and secondary lymphoid organs (Whitfield-Larry et al., 2014), while MDSC reduced in pancreatic islets, which was similar to Fu’s research (Fu et al., 2012) that indicated a negative correlation between MDSC in the islets and the progression of diabetes. The decrease of MDSC in the islets may be one of the reasons for the failure to salvage islet inflammation. After STZ injection, the number of MDSC in the peripheral blood increased significantly from day 3 and continued to increase to day 24, after which it remained stable at approximately twice of the normal control group (Venneri et al., 2015). Afterward, in the fourth week, the proportion of MDSC in the peripheral blood experienced the change from low to high, which may be due to the differentiation of MDSC (Hsieh et al., 2018). As for in the bone marrow, initially, the number of MDSC appeared to decrease and subsequently (about 2 weeks later) return to normal levels (Furman, 2015; Hsieh et al., 2018; Kim et al., 2018). In addition, a significant increase was noted in the number of MDSC in other organs. Increased number of MDSC in the spleen, bone marrow, kidney and pancreatic lymph nodes (PLN) was observed 3 weeks following STZ treatment (Gao et al., 2013; Hsieh et al., 2018). PMN-MDSC accounted for a large proportion of these cells in the bone marrow, whereas the PMN/M ratio was decreased (Gao et al., 2013; Kim et al., 2018; Li et al., 2021). In the PLN, the ratio of M-MDSC was decreased 15 days following STZ treatment (Carlos et al., 2017). MDSC in the peripheral blood of patients with T1D were also shown to be significantly higher than those of normal healthy volunteers. In an early study, MDSC (mainly M-MDSC) expansion was documented in the peripheral blood of T1D patients (Whitfield-Larry et al., 2014). Similar results were obtained in the blood of patients with diabetic nephropathy (Hassan et al., 2018). Also, it was noted that the frequency of M-MDSC in the group with HbA1c >7.5% was significantly higher (Skyler, 2004). However, a slight decrease in the M-MDSC and a significant increase in the PMN-MDSC of CD14^−^ were also indicated in the peripheral blood of patients with T1D (Hassan et al., 2018).

In T2D, leptin-deficient ob/ob mice and leptin receptor-deficient db/db mice can be used as the rodent model of spontaneous type 2 diabetes (Wang et al., 2014; Al-Awar et al., 2016; Todd, 2016). The frequency of MDSC in the spleen and the peripheral blood of db/db mice increased (Wang T. et al., 2018), and the proportion of MDSC in the blood was positively correlated with the fast blood glucose value in the ob/ob mouse model. Despite these findings, the proportion of MDSC in the bone marrow did not change. The number of MDSC in the spleen, fat and liver of peripheral organs significantly increased in the T2D mouse models (Xia et al., 2011). As for the T2D patients, MDSC exhibited a statistical increase in peripheral blood samples, based on different clinical sample sizes (Wang T. et al., 2018; Fernández-Ruiz et al., 2019; Islam et al., 2020). Further evidence suggests that the absolute number of M-MDSC was increased in the peripheral blood of patients with T2D compared with that noted in obese normal glucose volunteers (Friedrich et al., 2019).

Therefore, both in the animal model of diabetes and in the blood samples of clinical diabetic patients, total MDSC cells showed an increase, while the performance of the two subsets of MDSC is not uniform, giving us a better understanding of the diabetic pathological process.

## The Recruitment of MDSC in the Development of Diabetes

**FIGURE 1:**
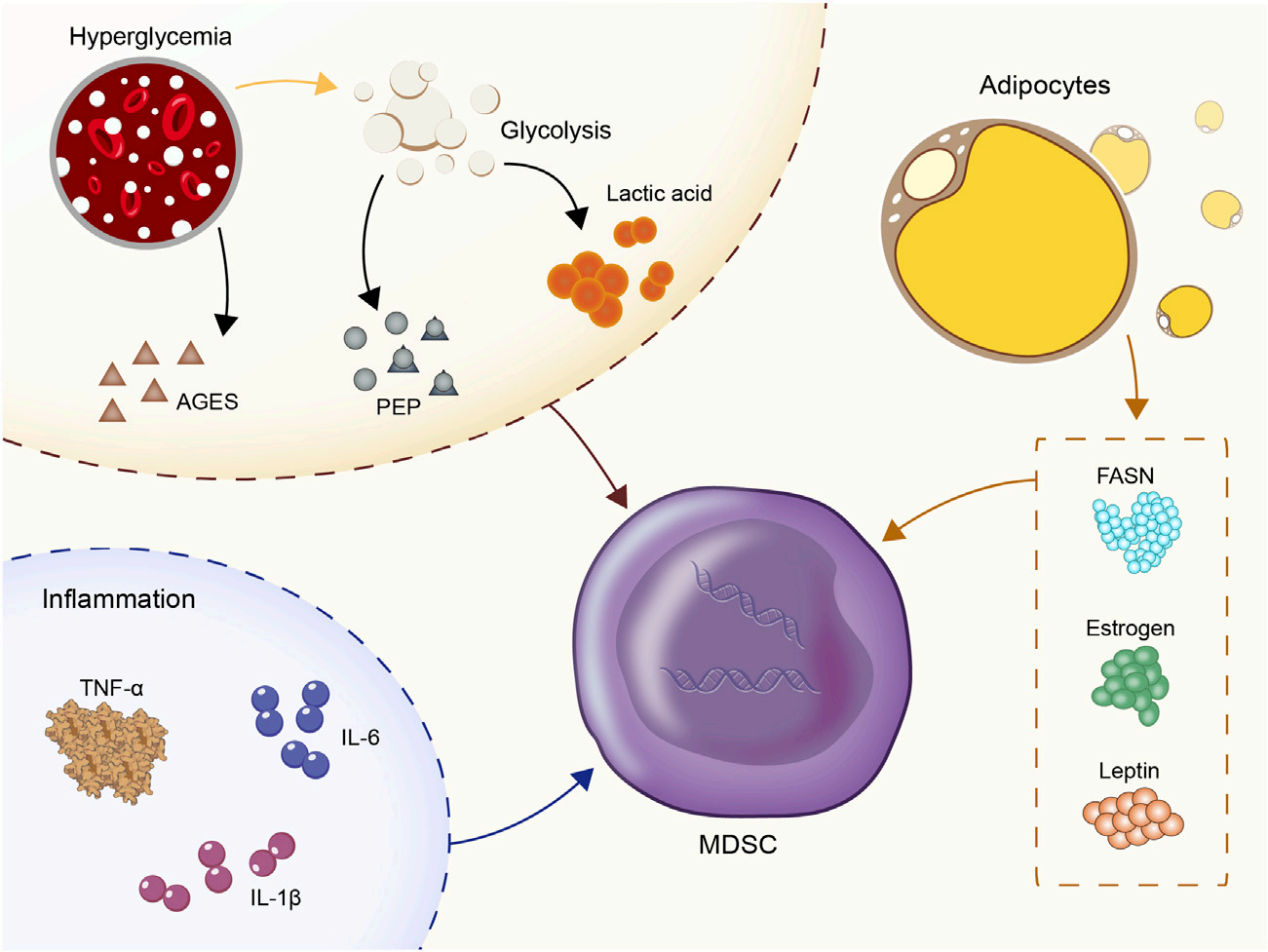
Four categories of molecules that recruit MDSC: Hyperglycemia and glycolysis products; inflammatory cytokines: IL-1β, IL-6, TNF-α; CC- chemokine ligand 2 (CCL2); estrogen, fatty acid synthase (FASN), and leptin.

The mechanism of MDSC aggregation in the diabetic microenvironment has not been fully investigated with regard to the induction of their recruitment. In the present study, we summarized four categories of molecules that recruit MDSC (Figure 1).

### The Role of Hyperglycemia and Glycolysis Products in MDSC Recruitment

Hyperglycemia and glycolysis products are the most prominent characteristics of the diabetic microenvironment. *In vitro* experiments showed that the number of MDSC derived from the bone marrow of normal mice was significantly increased in an environment containing high glucose (30M) (Li et al., 2021). *In vivo*, central carbon metabolism and bioenergetic kinetic models were used to confirm that high glycolysis is related to the maturation of MDSC (Goffaux et al., 2017). In human triple-negative breast cancer, upregulation of glycolysis also reduced the apoptotic activity of MDSC via the reduction of the levels of reactive oxygen species (ROS) produced by MDSC (Jian et al., 2017). Moreover, the subgroup CD11b^+^Ly6G^low^CD205^+^ of PMN-MDSC was heavily dependent on glucose uptake (Fu et al., 2021). High-flow and high-glucose glycolysis also lead to the rapid accumulation of the glycolytic process products and the TCA cycle products in the peripheral blood (Hui et al., 2017; Guasch-Ferré et al., 2020; Soto-Heredero et al., 2020). A positive correlation was noted between the glycolytic product lactic acid and the incidence of diabetes in several epidemiological studies (Crawford et al., 2010; Juraschek et al., 2013a; Juraschek et al., 2013b). Exogenous lactic acid was shown to increase the production of MDSC following stimulation by the granulocyte colony-stimulating factor (G-CSF) and interleukin-6 (IL-6) *in vitro* (Husain et al., 2013; Salminen et al., 2019). This in turn stimulated the immunosuppressive properties of MDSC. In addition, the glycolysis metabolite phosphoenolpyruvate (PEP), which is an essential antioxidant, prevented excessive production of ROS, thus contributing to the survival of MDSC (Jian et al., 2017). The advanced glycation end products (AGEs) are produced by non-enzymatic reactions between proteins and carbonyl compounds and are rapidly increased in hyperglycemia. The receptor for advanced glycation end-products (RAGE) is the receptor of AGEs (Prasad and Mishra, 2018). The expansion of MDSC mediated by the AGEs-RAGE interaction was reported in both cancer and acute myocardial infarction models (Yao et al., 2015; Huang et al., 2019). Therefore, the interaction of AGEs-RAGE in MDSC may be one of the reasons for the increase noted in the percentage of MDSC in the peripheral blood in patients with diabetes and poor long-term disease control.

### The Role of Inflammatory Cytokines in MDSC Recruitment

As an inflammatory microenvironment, diabetes is characterized by 2-3-fold elevated concentrations of tumor necrosis factor (TNF), IL-6 and C-reactive protein (CRP) in the peripheral blood. Inflammatory cytokines are active recruitment factors for MDSC. In T2D, blood glucose enhanced the expression of IL-1β in islets *ß* cells (Maedler et al., 2002) and the increase of low-density lipoprotein enhanced the expression of triggered IL-1β gene expression via TLR4 engagement (Masters et al., 2010). The signaling pathways, such as JNK and NF-kappa B, were also involved in the development of insulin resistance (Matulewicz and Karczewska-Kupczewska, 2016; Solinas and Becattini, 2017; Bako et al., 2019). IL-1β is regulated by the IL-1RI/NF-κB pathway and is a tumor-related factor leading to MDSC amplification and migration (Cervantes-Villagrana et al., 2020; Kuo et al., 2021). Previous studies showed a positive correlation between IL-1β and MDSC frequency in 4T1 breast cancer, lung cancer, advanced melanoma and other tumors (Bunt et al., 2006; Shi et al., 2017; Cervantes-Villagrana et al., 2020). IL-1β upregulates cyclooxygenase-2 (COX-2), which encodes prostaglandins that mediate MDSC transmission. In addition, the direct effect of IL-1β and its target gene products was assessed on the expansion of the MDSC library. It was shown that IL-1β further induced CC- chemokine ligand 2 (CCL2) (Guo et al., 2016) to amplify MDSC indirectly in macrophages and tumor cells.

Similarly, the increase in IL-6 levels in type 2 diabetes is mainly caused due to the higher rate of obesity and the formation of the inflammatory microenvironment (Pradhan et al., 2001; Rehman et al., 2017; Landers-Ramos et al., 2019). In malignant melanoma, squamous cell carcinoma (SCC), hepatocellular carcinoma (HCC), ovarian and bladder cancers, the expression levels of IL-6 were found to be positively correlated with the number of MDSC (Meyer et al., 2011; Chen et al., 2014; Bjoern et al., 2016; Lin et al., 2017; Wu et al., 2017; Xu et al., 2017; Yang et al., 2017; Tsai et al., 2019). A previous study conducted in melanoma demonstrated that IL-6 could upregulate the expression levels of CCR5 in MDSC through the STAT pathway, thus promoting their recruitment in the tumor microenvironment (Weber et al., 2020). The IL-6 signal transduction pathway activated STAT3 in MDSC in order to assist its immunosuppressive function, including increased synthesis of Arg-1 to consume I-Arginine in the microenvironment (Vasquez-Dunddel et al., 2013), which in turn promoted the expression of NOX2 and increased the concentration of ROS (Corzo et al., 2009; Chen et al., 2014) by releasing higher levels of NO (Xu et al., 2017). In addition, IL-6 downregulated the MHC-II class of myeloid cells and accelerated metastasis to more immature myeloid cells in tumors (Beyranvand Nejad et al., 2021).

The pro-inflammatory cytokine TNF-α was involved in the pathogenesis of diabetes by causing an increase in insulin resistance and a significant elevation in insulin levels in the diabetic microenvironment (Akash et al., 2018; Alzamil, 2020). Other TNF family members also promoted MDSC survival and increased MDSC aggregation by upregulating the expression levels of the cellular FLICE-inhibitory protein (c-FLIP) and by inhibiting caspase-8 activity (Zhao et al., 2012).

The increase in the expression levels of the classical inflammatory markers, JNK and the IκB kinase *ß* (IKKβ) played a role in promoting insulin resistance in the pathogenesis of diabetes (Yuan et al., 2001; Hirosumi et al., 2002; Cai et al., 2005). Moreover, these signaling pathways were also the cause of MDSC dilatation in the bone marrow (Flores et al., 2017; Guha et al., 2019). It is deduced that inflammation is very likely to be the driving force for promoting the survival of MDSC in the diabetic environment.

### The Recruitment of CCL2 on MDSC

The recruitment of MDSC is often dominated by the chemokine family members. For example, in cancer, chemokine (C-C motif) ligand (CCL) 2 and its receptors, chemokine (C-C motif) receptors (CCR) 2, 4, and 5 played a crucial role in attracting M-MDSC (Zhang et al., 2010; Chang et al., 2016; Korbecki et al., 2020; Gu et al., 2021). In endometriosis, CXCL1, 2 and 5 were expressed in the lesion site and the interaction of CXCR2 with MDSC facilitated the induction of a large number of MDSC (Zhang T. et al., 2018). Similarly, an elevated level of CCL2 was noted in plasma and eye fluids of patients with diabetic retinopathy (Maedler et al., 2002; Koleva-Georgieva et al., 2011; Koskela et al., 2013). We have reason to assume that the increased expression levels of CCL2 in the diabetic microenvironments may play a role in recruiting MDSC.

### The Role of Estrogen, Fatty Acid Synthase and Leptin in MDSC Recruitment

In particular, patients with type 2 diabetes have a high rate of obesity. Increased estrogen levels, fatty acid synthase (FASN) and leptin in the metabolic environment of obese patients were involved in the recruitment of MDSC. Obesity was associated with increased estrogen production by converting androgens to adipocytes through aromatase (Sasano et al., 2009). Estrogen is an important growth factor that stimulates the production of granulocytic monocytes in the bone marrow. Excessive estrogen production could lead to hematopoietic dysfunction, which in turn inhibited the production of mature dendritic cells from the bone marrow (Carreras et al., 2008). It was believed that excessive estrogen in obese individuals with type 2 diabetes might be a factor in recruiting MDSC. FASN is the key to adipogenesis in obesity (Berndt et al., 2007), which might induce MDSC aggregation and M2 macrophage differentiation via activation of the COX-2 pathway (Obermajer et al., 2011). The increased secretion of leptin by the adipose tissue in type 2 diabetes was shown to enhance the aggregation and immunosuppressive ability of MDSC (Clements et al., 2018).

The significant increase in the MDSC recruitment factors in the presence of the aforementioned diabetic microenvironment seems to explain the increase in the percentage of MDSC in these diabetic conditions. However, it is worth noting that complex differences exist with regard to the specific microenvironment in different diseases, and the response of the MDSC thus may differ with regard to these recruitment factors. With the exception of hyperglycemia, advanced glycolysis products, glycolysis and leptin, no specific studies have examined the role of chemokines in recruiting MDSC in the diabetic microenvironment up to now.

## The Interaction of MDSC With Innate Immune Cells

During the development of T1D, the classically activated macrophages release the pro-inflammatory cytokines IL-1β, IL-6, TNF-α, IFN-γ, IL-12, IL-17, and NO. It was reported that the number of macrophages in draining lymph nodes and joints of CIA mice was significantly decreased following treatment with MDSC (Zhang et al., 2014), indicating MDSC and macrophages may be converted to each other. Macrophages can be divided into pro-inflammatory (M1) and anti-inflammatory (M2) type cells. In obese subjects, Gr-1 cells induced the differentiation of macrophages into insulin-sensitive cells, which were alternately termed activated M2 macrophages (Xia et al., 2011). Intraperitoneal injection of Brazilian propolis ethanol extract (PEE) caused a direct stimulation of the transdifferentiation of cultured M1 macrophages into MDSC (Kitamura et al., 2018). The role of macrophages in diabetic wounds will be discussed below.

Dendritic cells (DCs) are important innate immune cells and professional antigen-presenting cells. During the pathogenesis of diabetes, CD4^+^T cells induced DCs to effectively stimulate CD8^+^T cells. The proportion of iNOS^+^ DCs in patients with diabetes was significantly increased and these cells were usually activated (Gajovic et al., 2018). As immature myeloid immunosuppressive cells, MDSC has the potential to differentiate into DCs. Tumor-derived factors redirected the differentiation from immune-promoting DCs to tolerable MDSC, which could be an immunological marker of cancer (Rodríguez-Ubreva et al., 2017). Concomitantly, myeloperoxidase-driven lipid peroxidation in PMN-MDSC inhibited antigen cross-presentation of DCs (Ugolini et al., 2020). M-MDSC directly targeted subsets of DCs and produced NO in a NOS2-dependent manner to rapidly lyse conventional and plasma cell-like DCs (cDC, pDC). This process indirectly inhibited the effector T cell response (Ribechini et al., 2019). Synovial fluid (SF) cells in arthritic joints of PGIA mice had the characteristics of MDSC and could inhibit the differentiation of DCs by reducing the expression levels of MHCII and CD86 (Egelston et al., 2012). The interference of MDSC on the antigen presentation function of DCs may be one of the key mechanisms to unravel the immune-mediated development of diabetes.

## The Modulation of MDSC on Adaptive Immune Cells in Diabetes

The pathogenesis of T1D is based on the attack of CD4^+^ and CD8^+^T cells on islet *ß* cells. The cells establish a connection that triggers the release of perforin and granzyme B from cytolytic granules inside the T cells. A hole is made in the cell to allow granzyme B to enter the cytoplasm, release cleaved caspase-3 and activate the apoptotic pathway (Pirot et al., 2008). CD4^+^T cells assist CD8^+^T cells, B cells and DCs while secreting cytokines to attack islet *ß* cells (Burrack et al., 2017). Recent studies showed that CD4^+^T cells also directly attacked certain MHCII-positive *ß* cells (Zhao et al., 2015). MDSC suppress CD4^+^ and CD8^+^T cells by direct contact, consuming I-Arginine and releasing NO and ROS. The inhibition of T cells was further confirmed to be Ag-dependent and MHCII-restricted (Yin et al., 2010). Moreover, the adoptive transfer of diabetic CD4^+^T cells and MDSC to NOD/SCID mice significantly reduced the incidence of diabetes caused by single-transferred CD4^+^T cells, further clarifying that MDSC delayed diabetes by inhibiting T cells. However, the immunosuppressive ability of MDSC in the diabetic microenvironment could also shuttle between normal and tumor environments. MDSC of mice with allogeneic breast tumors had a more robust ability to inhibit T cell proliferation and Treg cell expansion. Adoptive metastasis delayed the onset of diabetes in NOD/SCID mice (Yin et al., 2010; Wang T. et al., 2018).

The same finding was also confirmed in T1D patients. Bone marrow mesenchymal stem cells from T1D patients inhibited T cell proliferation in a contact-dependent manner, but their immunosuppressive ability was enhanced by cytokine induction *in vitro* (Whitfield-Larry et al., 2014). The anti-diabetic effect of MDSC may be further enhanced by inducing cytokine expression in patients with diabetes. Concomitantly, different subsets of MDSC play different roles, which are similar to those noted in the tumor and EAE microenvironments. In addition, it was reported that M-MDSC were the specific cell group that mainly exerted its immunosuppressive ability to T cells in the T1D microenvironment (Whitfield-Larry et al., 2014). Although their activity was weaker than that noted in tumors (M-MDSC: T cells = 1:4), the M-MDSC effectively inhibited T cell proliferation and decreased the expression levels of CD3-ζ chain cytokines in T cells at a 1:1 ratio compared with TGF-β, thus enhancing T cell tolerance (Grohová et al., 2020). Due to the strong immunosuppressive ability of the MDSC on T cells, the development of diabetes may proceed due to the reduced immunosuppression.

The roles played by CD4^+^ and CD8^+^T cells are also noted in T2D pathogenesis, while the increased levels of MDSC in the db/db mice exhibited an immunosuppressive effect on CD4^+^T cells. MDSC also releases IFN-γ and iNOS to inhibit the function of CD8^+^T cells. It was shown that the increased accumulation of MDSC enhanced insulin response, while depletion of MDSC significantly reduced glucose tolerance and insulin sensitivity and this process was related to the ability of MDSC to inhibit inflammation (Xia et al., 2011). However, it is not clear whether MDSC maintains its inhibitory properties *in vivo*. We speculated that the frequency of M-MDSC in the peripheral blood might be too low to exert its inhibitory potential, or the immunity cannot be entirely suppressed since the local microenvironment affects MDSC.

B cells are the critical antigen-presenting cells of CD4^+^ and CD8^+^T cells. Autoantibodies may not directly cause pathology but aid in capturing restricted antigens and promote T cell initiation by spreading the autoimmune response (Serreze et al., 1998). The presence of B cells in islet lesions was positively correlated with rapid *ß* cell destruction, early-onset time and the development of more aggressive diseases (Morgan et al., 2014; Wedgwood et al., 2016; Greenbaum and Lord, 2020). If B cells could not secrete autoantibodies, the incidence and penetrance of T1D would be significantly reduced (Wong et al., 2004). The transfer of serum autoantibodies stimulated the activation of CD4^+^T cells (Silva et al., 2011). Clinically, patients with eliminated B cells due to treatment with anti-CD20 (rituximab) reduced their need for exogenous insulin for a long time period (Pescovitz et al., 2009). In the SLE mouse model, PMN-MDSC induced the activation of IFN-I signal transduction in B cells. MDSC treatment reduced immunoglobulin production of autologous B cells in a dose-dependent manner in mice with rheumatoid arthritis (Crook et al., 2015). Previous studies on mouse AIDS models showed that MDSC inhibited B cell responses by releasing reactive oxygen/nitrogen species and TGF-β (Rastad and Green, 2016). During mouse tumor progression, MDSC reduced IL-7 and STAT5 expression levels as well as the B cell response to suppress B cell proliferation in an arginase-dependent manner that required cell-to-cell contact *in vitro* (Wang Y. et al., 2018). In contrast to these observations, human M-MDSC effectively inhibited the proliferation and function of human B cells in a non-contact manner via the release of NO, Arg1 and IDO *in vitro* (Jaufmann et al., 2020). In an experiment in which anti-CD20 antibodies depleted B cells in NOD mice, MDSC amplified, inhibited the function of CD4^+^ and CD8^+^T cells through cell-to-cell contact and released IL-10 and NO, ultimately inhibiting the development of type 1 diabetes in mice (Hu et al., 2012).

As an essential component of the peripheral immune tolerance, Tregs stably express Foxp3, inhibit CD4^+^ and CD8^+^ T cells, and secrete IL-10 and TGF-β (La Cava, 2009; Mohr et al., 2019). The change in the number of Tregs is controversial following the development of diabetes. The number of Tregs in the pancreatic lymph nodes of mice was abnormally increased when the animals approached the onset of the disease, whereas the ratio of Treg/Teff in the islets was decreased. This finding could be attributed to a decrease in the number of Tregs or to resistance induced by Treg cell-mediated inhibition (Tang et al., 2008). Tregs produced more IL-10 in 8-week-old mice than 16-week-old mice and delayed the pathogenesis of T1D induced by adoptive transfer effector cells, indicating that the function of Tregs decreases with age (Gregori et al., 2003). Similarly, Treg defects were reported in T1D patients. Further studies demonstrated that *in vitro* expansion and re-infusion of Tregs into diabetic NOD mice effectively reversed spontaneous and adoptive metastasis of T1D (Tang et al., 2004). Clinically, the *in vitro* expansion of Tregs and the subsequent reinfusion resulted in the C-peptide being stable for more than 2 years as determined in Phase I clinical trials (Bluestone et al., 2015). MDSC were shown to promote the expansion of Tregs in the tumor microenvironment (Li F. et al., 2018; Wu et al., 2019; Sieminska and Baran, 2020; von Arx et al., 2020). CD115^+^ MDSC (mainly M-MDSC) in the diabetic microenvironment expanded Foxp3^+^ Tregs in a TGF-β dependent manner both *in vivo* and *in vitro* (Wu et al., 2015; Zhao et al., 2016), whereas the expansion of Tregs was Ag-dependent and MHCII-restricted (Yin et al., 2010).

As the increase in the number of MDSC in the diabetic environment, its immunosuppressive ability to T cells was lower than that in the tumor environment (Gajovic et al., 2018). As an autoimmune-mediated disease, the network of immunosuppression between MDSC and the immune cells may happen to inhibit the pathogenesis of diabetes. MDSC exerts a certain degree of immunosuppressive ability in T1D, while the ability to inhibit inflammation and immunosuppression in T2D slows down the progression of diabetes. Therefore, regulating the frequency and activity of MDSC may become an expected method for the treatment of diabetes.

## MDSC is a Candidate Target for the Treatment of Diabetes

### Stem Cell Therapy

Stem cell therapy has been a research hotspot in recent years. As a newly discovered subgroup of MDSC, f-MDSC are CD33^+^IL-4R α^+^ fibrous cells differentiated by umbilical cord blood progenitor cells cultured with FDA-approved cytokines (rh-GM-CSF and rh-G-CSF). These cells are cultured for 4 days and display a fibroblast-like shape. They are also characterized by cytoplasmic elongation, nuclear nucleoli, phagocytic extension and high adhesion to plastic (Mazza et al., 2014). F-MDSC have been shown to produce IDO following their interaction with activated T cells in NOD/SCID mice in order to promote Tregs differentiation and reduce blood glucose to normal levels for therapeutic purposes (Zoso et al., 2014). Human umbilical cord mesenchymal stem cells (Huc-MSCs), which are widely used in NOD mice, can inhibit the differentiation of MDSC by secreting the soluble factors COX2/PGE2 and IFN-β and enhance their inhibitory ability on immune cells so as to achieve effective therapeutic effects against diabetes (Qi et al., 2020). Based on the aforementioned findings, the metastasis of MDSC, f-MDSC, or the transfer of Huc-MSCs may possess the enhanced inhibitory ability on immunity and demonstrate the therapeutic effect on diabetic mice (Yin et al., 2010; Xia et al., 2011; Torbica et al., 2019; Ren et al., 2021). MDSC-related stem cells also may be a promising treatment for diabetes.

### Anti-Gr-1 Antibody

The use of antibodies against the molecular markers of MDSC was initially intended to deplete the MDSC of the subject. A one-time intravenous injection of anti-Gr-1 only temporarily reduced MDSC in the body, followed by a long-term increase in Gr1^+^ CD11b^+^cells, which was similar to the findings noted in the tumor microenvironment (Ma et al., 2012). When the anti-Gr-1 antibody (0.25 mg/mice) was injected intravenously in NOD mice, MDSC depletion lasted only 4 days and the number of MDSC was significantly higher than that in the control group noted from the 7th to the 17th day following treatment (Hu et al., 2012). These findings suggest that short-term injection of anti-Gr-1 antibody may induce a long-term compensatory increase.

### Functional Molecule Inhibitor

Rapamycin is a specific inhibitor of mTOR. It can trigger the inhibition of mTORC1, which leads to the increase of Tregs and MDSC. Rapamycin reduced the phosphorylation of S612 (insulin receptor substrate-1) in adipose, muscle and liver tissues, which inhibited the degradation of IRS-1 and improved insulin sensitivity in mice. Concomitantly, rapamycin also adjusted the classification of MDSC, increased the number of PMN-MDSC in fat and liver tissues and in the blood and reduced M-MDSC, thereby reducing inflammation (Pederson et al., 2001; Makki et al., 2014). It was indicated that the mTOR inhibitor INK128 could inhibit the differentiation of M-MDSC into M1 pro-inflammatory macrophages, thus reducing inflammation and promoting diabetic wound healing (Li et al., 2021). Knockout of the C3 complement gene significantly promoted the immunosuppressive ability of MDSC. In STZ-induced T1D mice, MDSC were highly activated and suppressed T cells in order to regulate Treg cells via TGF-β secretion. A similar effect was achieved by using the complement activation inhibitor FUT-175 (Gao et al., 2013).

IL-17^-/-^ mice resisted STZ-induced diabetes by increasing the percentage and number of MDSC in the spleen and enhancing their immunosuppressive ability (Tong et al., 2015). Therefore, it was expected that IL-17 inhibitors could also play an anti-diabetic effect. In addition, K118 is an inhibitor of PISHIP1, which was shown to target SHIP1 in MDSC derived directly from visceral adipose tissues and increase the number of MDSC and improve blood glucose control and insulin sensitivity. K118-treated mice demonstrated no harmful side effects in the lung, small intestine, or other organs and in the bone mineral density (Srivastava et al., 2016). Although the number of activated CD4^+^T and CD8^+^T cells was decreased, MDSC was determined by the positive expression of IL4aR and Arg and the T cell inhibitory effect of MDSC was not directly measured in that study.

### Diet

Dietary polyunsaturated fatty acids rely on STAT3 signaling to increase MDSC and ROS production to enhance their immunosuppressive ability, which was almost entirely reversed by application of the STAT3 inhibitor JSI-124 (Yan et al., 2013). In tumors, MDSC use fatty acid oxidation as their energy supply and the tumor microenvironment can cause upregulation of the expression of enzymes critical to fatty acid oxidation, thus increasing the inhibitory ability of MDSC. These findings were confirmed both in mice and humans (Hossain et al., 2015). Therefore, a new theoretical basis has been deduced for dietary recommendations to increase the intake of polyunsaturated fatty acids for T2D. Nevertheless, as a sensitive cell group, the effect of comprehensive food intake on MDSC may change. In a recent experiment that examined atherosclerotic subjects, the number and proportion of MDSC in the bone marrow of high-fat diet mice were both decreased following co-administration with omega-3 polyunsaturated fatty acids, flavanols, and phytosterols (Moss et al., 2021). However, the number and function of MDSC in the blood are not currently known under these feeding conditions. Additional research is thus required to explain these findings.

Brazilian propolis is a resin mixture of African honey bee saliva and wax mixed with plant exudates (Kumazawa et al., 2003; Bankova et al., 2014). It has been widely used in folk medicine due to its anti-inflammatory, anti-viral, analgesic and metabolic effects (Tsuchiya et al., 2018; Al-Hariri and Abualait, 2020). Following intraperitoneal injection, Brazilian propolis was shown to induce visceral adipose tissue and intraperitoneal MDSC production in mouse models, which exerts an anti-inflammatory effect and improves the severity of T2D (Kitamura et al., 2018). Oral administration of PEE should be recommended to assess the induction of the stimulation of human MDSC and the potential side effects.

Intestinal flora plays a vital role in the pathogenesis of diabetes and has been a research hot spot in recent years (Dolpady et al., 2016; Ghaisas et al., 2016; Jia et al., 2017; Henschel et al., 2018; Mullaney et al., 2018). The IgM purified from the serum of normal mice can maintain the normal Bacteroides: Firmicutes ratio and reverse the pathogenesis of diabetes in NOD mice following administration by intraperitoneal injection. The number of Tregs and MDSC in mice treated with IgM was significantly increased. Oral feeding exhibited a certain effect on this process (Chhabra et al., 2018). This also should be used as another convenient and feasible way to increase the number of MDSC.

### Other Therapies

Worm infection and its antigens, such as soluble (TCS) or excretory/secretory (TCES) antigens derived from Taeniasolium can increase the number of MDSC in T1D mice. Intravenous injection of dichloromethylene diphosphonate (clodronate) encapsulated in liposomes depleted macrophages but increased the number of MDSC and their subtypes (Espinoza-Jiménez et al., 2017).

Various factors can affect the proliferation and recruitment of MDSC and related studies have been performed in the tumor microenvironment. Among them, HIF-1α, which is decreased due to the instability of the HIF protein in the diabetic microenvironment, is a strong chemokine of MDSC and regulates their function. HIF-1 can bind to the erythropoietin gene promoter during hypoxia and form heterodimerization of HIF-1α and HIF-1β. Insulin signaling upregulates HIF-1α through phosphorylation of PI3K and MAPK. However, diabetic patients had impaired insulin signaling due to insulin resistance. The levels of HIF-1 *α* decreased and the lack of HIF-1α further weakened the function and survival of *ß* cells, forming a vicious circle (Cheng et al., 2010). The induction of hyperglycemia enabled the stimulation of the degradation of HIF-1α by 2-methyl Glyoxal and inhibited its transcriptional activity. 2-methyl Glyoxal inhibited the formation of the HIF-1α-HIF-1β dimer (Bento and Pereira, 2011). In obese diabetic subjects, a decrease in succinic acid was caused by fatty acid metabolism and an increase in HIF-1α protein hydrolysis (Dodd et al., 2018). HIF-1α was shown to be effective in increasing the number and function of MDSC in the tumor microenvironment. HIF-1α also activated glucose transporter-1 (Glut-1) to promote glycolysis, thus exerting the effect of glycolysis on MDSC (Choi, 2017). HIF-1α yet induced the expression of nucleoside diphosphate hydrolase 2 ENTPD2/CD39L1 in order to consume extracellular ATP, which in turn promoted the maintenance of MDSC (Chiu et al., 2017).

It is known that metformin can reduce the phosphorylation levels of STAT3 and inhibit the expression of CD73/CD39 on MDSC by activating AMPK and inhibiting the HIF-1α pathway to exert an inhibitory effect on MDSC (Li L. et al., 2018; Xu et al., 2019). However, it is unclear whether metformin has a special regulatory effect on MDSC in a diabetic microenvironment.

## The Role of MDSC in Diabetic Complications

In several complications of diabetes, specific organ damage leads to the corresponding increase in the recruitment and activity of MDSC. MDSC play different functions in different complications. Among them, the complications caused by MDSC microangiopathies, such as diabetic retinopathy, diabetic nephropathy, and diabetic refractory wounds are prominent. These conditions will be discussed separately below.

### Diabetic Nephropathy

Diabetic nephropathy is the leading cause of the end-stage renal disease (Li et al., 2016). The pathogenesis mainly lies in the fibrosis caused by the accumulation of extracellular matrix proteins in the glomerular mesangial interstitium (Chang et al., 2014). The number of MDSC in the kidney was increased (Xing et al., 2017), and the adoptive transfer of MDSC induced by cytokines reduced fibronectin levels in the glomerulus and resulted in a normal glomerular filtration rate (Hsieh et al., 2018). PMN-MDSC in the kidneys of patients with T2DN might not be sufficient to maintain renal function, resulting in compensatory anti-inflammatory failure of the kidney (Duran-Salgado and Montserrat, 2014; Islam et al., 2020). The increased number and enhanced anti-inflammatory ability of PMN-MDSC may be one of the therapeutic targets for diabetic nephropathy.

### Diabetic Atherosclerosis

Atherosclerosis (AS) is the leading cause of coronary heart disease, cerebral infarction and peripheral vascular disease (Lusis, 2000). Diabetes accompanied by high fat and an inflammatory environment is a fundamental cause of the development of atherosclerosis (Poznyak et al., 2020). The role of MDSC and their subgroups in AS is still controversial (Wang et al., 2015; Fernández-Ruiz et al., 2019). It was demonstrated that MDSC increased about two-fold in the bone marrow of AS model mice, where M-MDSC increased in proportion and PMN-MDSC decreased, and the inhibitory activity of M-MDSC was enhanced (Foks et al., 2016). This was consistent with the phenomenon observed in peripheral blood samples of AS patients (Wang et al., 2015). Moreover, the frequency of PMN-MDSC was negatively correlated with low-density lipoprotein cholesterol (Fernandez-Ruiz et al., 2019). It was proposed that the increase of MDSC in the bone marrow of depressed AS mice, especially PMN-MDSC, could increase neutrophil traps (NETS) and aggravate AS (Yamamoto et al., 2018). The opposing effects of the two subgroups of MDSC remind us to observe the role of MDSC in AS and score clear subgroups. In the LDLr^-/-^ model (Foks et al., 2016) and the ApoE^-/-^ murine model (Wang et al., 2020) fed on the Western-type diet (WTD, containing 0.25% cholesterol and 15% cocoa butter) diet (0.25% cholesterol and 15% cocoa butter), MDSC were shown to significantly slow down the disease process of AS after adoptive transfer. Also, the therapy for MDSC was regarded as one of the hopes for the treatment of atherosclerosis. It was proved that oral HSP60 reduced the development of AS by increasing the number of M-MDSC and enhancing its function, while subcutaneous HSP60 caused the opposite response (Hu et al., 2018). In addition, SBI-0206965, an inhibitor of autophagy, rapidly reduced MDSC and promoted the development of atherosclerosis (Wang et al., 2020). Therefore, the immunomodulation of MDSC and its subgroups may be regarded as a potential treatment of atherosclerosis.

### Diabetic Retinopathy

The pathogenesis of diabetic retinopathy lies in the structural disorder of microvessels. The main sign of the development of the non-proliferative type to a more advanced proliferative type is the proliferation of ocular neovascularization (Hendrick et al., 2015). Existing drugs are mainly focused on preventing neovascularization, such as the anti-VEGF drug ranibizumab (Chatziralli and Loewenstein, 2021). In the ocular humor of patients with DR, both levels of IL-6 and CCL2, which are important proliferative factors of MDSC, and the number and activity of myeloid cells were observed to be increased (Koleva-Georgieva et al., 2011; Koskela et al., 2013). MDSC play a role in stabilizing blood vessels in diabetic retinopathy (Liyanage et al., 2016; Villacampa et al., 2020). MDSC reduced retinal neovascularization in oxygen-induced retinopathy (Kataoka et al., 2011; Xu et al., 2018) and laser-induced choroidal neovascularization (Espinosa-Heidmann et al., 2003; Sakurai et al., 2003; Nagai et al., 2007). These actions of MDSC were different from those noted during tumor angiogenesis (Albini et al., 2018; Zhang T. et al., 2018; Yang et al., 2018; Lin et al., 2019; Rahma and Hodi, 2019; Dysthe and Parihar, 2020).

### Diabetic Refractory Wounds

The difficulty in wound healing of diabetic patients is caused by chronic inflammation, vascular endothelial injury, hypoxia, autonomic nervous dysfunction and decreased neuropeptide signal transduction (Noor et al., 2015). The diabetic foot ulcer is the most crucial reason for the amputation of patients with non-traumatic conditions (Everett and Mathioudakis, 2018). MDSC distribution could change due to the addition of the wounds. During the period from the inflammatory to the proliferative phase, a new round of proliferation of bone marrow MDSC was stimulated by the wound. MDSC led to peripheral distribution and targeted the wound (Mahdipour et al., 2011; Li et al., 2021). It was shown that the CD11b^+^Ly6C^hi^ cell group on the wound was rapidly transformed into CD11b^+^Ly6C^low^ cells within 1–2 days of wound formation. Subsequently, an additional CD11b^+^Ly6C^hi^ cell group flowed into the wound on the 3rd-4th day sequentially (Kimball et al., 2018). Nevertheless, MDSC were retained at a higher density explicitly in the assumed granulation tissue area of the wound. They were associated with endothelial cells at the injury site and their frequency was higher than that of non-diabetic mice (Torbica et al., 2019). The role of MDSC in diabetic wounds was examined, suggesting that immature myeloid cells could impair diabetic wound healing, while the use of G-CSF in db/db wounds could accelerate wound healing (Wicks et al., 2015). However, the positive effect of MDSC on wound healing was supported by various studies. The fact that the adoptive transfer of MDSC derived from the spleen (Mahdipour et al., 2011) or the bone marrow (Tong et al., 2014) of mice to diabetic wounds could assist wound healing has become an essential supporting basis. The number of blood vessels was analyzed in diabetic and non-diabetic mice by injecting bone marrow-derived MDSC into the wounds of diabetic and non-diabetic mice. The results demonstrated that MDSC benefited angiogenesis, whereas the diabetic microenvironment impaired their ability (Mahdipour et al., 2011). Besides, MDSC derived from the bone marrow of diabetic mice indicated decreased proliferation and differentiation, decreased chemotactic function, lower expression of VEGF and higher MMP-9 levels as determined by *in vitro* studies (Torbica et al., 2019). In addition, the recruitment of MDSC in wounds might be impaired by damaging the SDF-1/CXCR4 axis (Tong et al., 2014).

Abnormal differentiation of MDSC typing can also be noted in diabetic wounds. Bone marrow cells can be activated or polarized into different states related to Th1 and Th2 cytokines by the local microenvironment. These myeloid cells are termed classically activated (M1) or alternatively activated (M2) cells. The diabetic microenvironment affected the pedigree commitment of these progenitor cells, inhibited granulosa cell differentiation and promoted monocyte differentiation (Mahdipour et al., 2011). The M1 group on the diabetic wound was mainly composed of M-MDSC. In the subsequent stage of healing, macrophages were transformed into M2 cells in the non-diabetic wound and retained a large amount of pro-inflammatory M-MDSC in the diabetic wound (Mahdipour et al., 2011; Bannon et al., 2013). The transformation of MDSC to PMN-MDSC (like overexpressing Hoxa3) resulted in significant induction of neovascularization in diabetic wounds.

**FIGURE 2:**
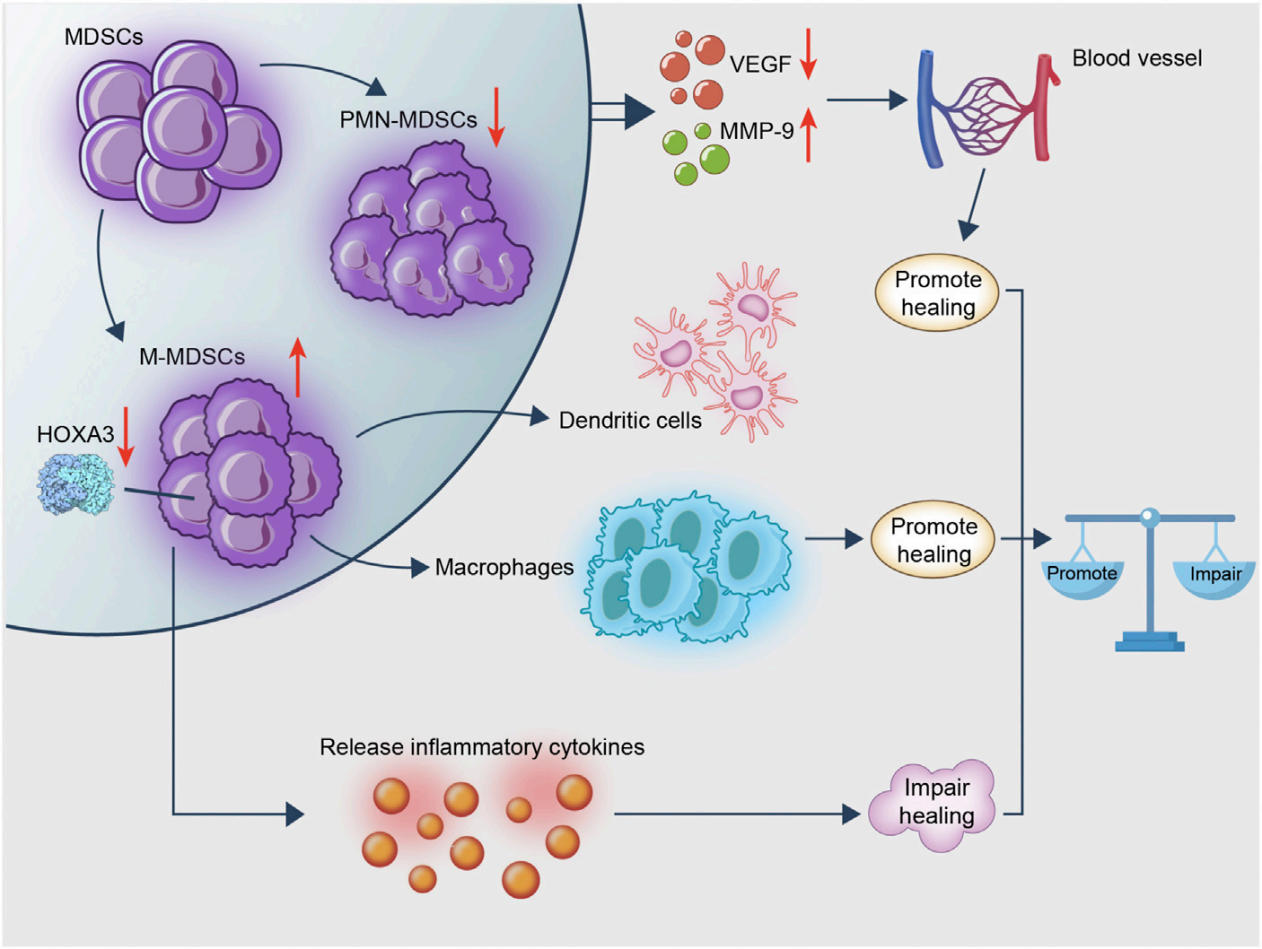
MDSC is rapidly transformed into macrophages following recruitment to the wound. This process exerts an anti-inflammatory effect and promotes wound healing. MDSC remains in diabetic wounds and affects angiogenesis, whereas diabetes hinders the recruitment of MDSC from the wound; the existence of an imbalance ratio of PMN/M-MDSC keeps the wound in an inflammatory microenvironment hindering wound healing. Therefore, MDSC promotes wound healing, whereas excessive M-MDSC impairs wound healing.

In view of these studies, it is suggested that MDSC may promote wound healing by their conversion to CD11b^+^Ly6C^low^cells, which in turn promote angiogenesis. However, the effect of the complex diabetic microenvironment on MDSC, such as the imbalance between the different types of MDSC, may impair wound healing (Figure 2).

## Summary and Conclusion

Altogether, diabetes is mainly divided into T1D and T2D. We summarized the variation in quantities, classification, activity and immunosuppressive ability of MDSC in T1D and T2D. The underlying roles in diabetes and potential MDSC-targeting diabetes treatment were assessed. The diabetes environment activates the one-time development of MDSC in the bone marrow and promotes the accumulation of MDSC in peripheral organs except for pancreatic islets. This is inseparable from the abundant MDSC recruiting factors, such as glycolysis products, inflammatory factors, CCL2, etc. MDSC exert a certain degree of immunosuppressive ability in T1D, while their ability to regulate inflammation and immunosuppression in T2D slow down the progress of diabetes. Therefore, many possibilities for the treatment of diabetes related to MDSC have been derived. In addition, the main pathology of diabetic complications focuses on the disorder of blood vessel formation and the inflammatory environment. The stable angiogenesis and immunosuppressive ability of MDSC should have a therapeutic effect on complications. However, the performance of MDSC subsets in diabetic complications differs widely. NETS developed by excessive PMN-MDSC in AS and pro-inflammatory excess of M-MDSC in diabetic refractory wounds aggravates the development of the disease. In-depth understanding of MDSC and its subsets, and intervention and adjustment according to different pathological characteristics, will gradually become the key to making good use of the double-edged sword of MDSC and personalized immunotherapy.

## Abbreviations

AS, Atherosclerosis; CCL2,CC, chemokine ligand 2; c-FLIP, FLICE-inhibitory protein; COX-2, cyclooxygenase-2; CRP, C-reactive protein; DPP-4, dipeptidyl peptidase-IV; e-MDSC, early stage MDSC; FASN, fatty acid synthase; GLP-1, glucagon-like peptide-1; G-MDSC, granulocytic MDSC; HCC, hepatocellular carcinoma; Huc-MSCs, Human umbilical cord mesenchymal stem cells; IFN-γ, interferon-γ; IKKβ,IκB kinase β; IL-6,interleukin-6; iNOS, inductible nitric oxide synthase; MDSCs, Myeloid-derived suppressor cells; NETS, neutrophil traps; NOD mice, non-obese diabetic mice; PEE, Brazilian propolis ethanol extract; PLNS, pancreatic lymph nodes; PMN-MDSC, polymorphonuclear MDSC; ROS, reactive oxygen species; SCC, squamous cell carcinoma; STZ, streptozotocin; T1D, type 1 diabetes; T2D, type 2 diabetes; TNBC,triple-negative breast cancer; TNF, tumor necrosis factor; UCB, umbilical cord blood.
